# Maternal perceptions about caesarean section deliveries and their role in reducing perinatal and neonatal mortality in the Upper West Region of Ghana; a cross-sectional study

**DOI:** 10.1186/s12884-019-2536-8

**Published:** 2019-10-11

**Authors:** Barnabas B. Naa Gandau, Benjamin D. Nuertey, Nana Ayegua Hagan Seneadza, Dominic Akaateba, Emmanuel Azusong, Judith Y. Yirifere, Herta B. Kankpeyeng, Edem M. A. Tette

**Affiliations:** 1 School of Medical Science, University for Development Studies, Tamale, Ghana; 2 Upper West Regional Hospital, Wa, Ghana; 3 Community Health Department, School of Public Health, University of Ghana, Accra, Ghana; 4 Public Health Department, Tamale Teaching Hospital, Tamale, Ghana; 5 Shai-Osudoku District Hospital, Dodowa, Ghana

**Keywords:** Caesarean section, Neonates, Maternal perceptions, SDG, Antenatal clinic

## Abstract

**Background:**

Maternal perceptions about caesarean section contribute to delayed presentation of women for emergency obstetric care. This increases the risks of perinatal and neonatal mortality and slows down the reductions needed to achieve the sustainable development goal (SDG) target of reducing neonatal mortality and ending new-born deaths. The aim of the study is to determine maternal perceptions about caesarean section deliveries and their role in reducing neonatal mortality at a regional and a district hospital in the Upper West Region of Ghana.

**Methods:**

This descriptive study was carried out at two hospitals in the Upper West Region, the most rural region in Ghana, between 15th January and 29th June, 2018. Maternal perceptions were examined among antenatal care attendants at the Upper West Regional Hospital (UWRH) and St Joseph’s Hospital Jirapa (SJH), a district hospital, using questionnaires administered by trained nurses.

**Results:**

Altogether, 416 completed questionnaires were obtained, comprising 206 from expectant women attending the UWRH and 210 from SJH. Although the majority of women in this study preferred spontaneous vaginal delivery (87.4%, *n* = 348) to caesarean section, most of the respondents (*n* = 281, 73%) indicated their willingness to have a caesarean section if necessary. The main reason for not wanting a CS was the long recovery time (51.8%, *n* = 148). Almost half of women interviewed, representing 45.1% (180) did not know or feel that CS can promote child survival and about a fifth, 21.6% (85) believed that CS can have adverse effects on child survival. Factors associated with poor perception of CS included, no formal education, age less than 19 years and no employment.

**Conclusion:**

Majority of women in this study had a positive attitude towards the uptake of CS if it becomes necessary. Lack of formal education, age less than 19 years and unemployment are associated with poor maternal perception of CS. Education to improve the perception of CS as a promoter of child survival is necessary and to discourage perceptions that it causes adverse perinatal or neonatal outcome particularly in at risk populations.

## Background

Neonatal and perinatal mortality in low and middle income countries is a matter of concern. This is because in the year 2015 alone, almost half (2.7 million) of the 5.9 million under-five deaths occurred during the neonatal period [1]. In addition, about 98% of global perinatal and neonatal mortalities are reported to occur in low and middle income countries [2]. In Ghana, for instance, the neonatal mortality rate in 2017 was estimated at 29 per 1000 live births [3, 4] and it represented 48% of under-five mortality (60/1000 live births). Meanwhile, the Sustainable Development Goals (SDGs) seek to tackle this problem with the aim of ending preventable deaths of new-borns and reducing neonatal mortality to at least as low as 12 deaths per 1000 live births by 2030 [5, 6].

It is estimated that, improved care at birth has the potential of preventing 1.3 million stillbirths within these regions by the year 2020, especially since the majority of the causes of stillbirths are preventable [7]. Therefore, since neonatal and perinatal deaths are closely related to the quality of care a woman receives during pregnancy and labour, accessibility of emergency obstetric care services including caesarean section when indicated is essential for reducing these deaths [8, 9].

Studies have shown that having a skilled attendant at delivery, and institutional delivery in general, has been associated with reductions in maternal, perinatal and neonatal mortality [10]. One of the critical advantages of institutional skilled delivery is the window of opportunity for surgical intervention when needed. Access to caesarean section is recognised as an essential component of obstetric care [11, 12] and, when medically justified, it is known to prevent perinatal and maternal morbidity and mortality [13]. However, maternal perceptions about caesarean section may influence the likelihood of mothers opting for institutional delivery which in turn affects access to this intervention, especially when it is most needed. It has been reported that caesarean section rates at population levels higher than 10% were not associated with reductions in maternal and new-born mortality rates [14, 15]. However, in Africa where the median caesarean section rate is estimated to be 8.8%, the risk of neonatal death is reported to be lower in settings having higher elective caesarean section rates [16]. The national average of caesarean section rates in Ghana is estimated at 12.8% but it ranges from 11.2–14.6 [17]; even then, there are wide disparities in access and uptake of caesarean section in urban and rural areas which may be partly related to perceptions about caesarean section.

There is a widespread belief that West African women have an aversion for surgical delivery and to have a CS is regarded as a reproductive failure [18–22]. Vaginal delivery is regarded as the ideal and a status symbol of womanhood; therefore, women who have had a CS might feel loss of the idealised birth they had hoped for, loss of part of their womanhood, and live in fear that other women may ridicule them [18–22]. A recent at study conducted in the Upper West Region described women’s perception of caesarean section delivery as highly problematic; and that CS delivery acts as a long term disease [23]. This was because the respondents felt that having a CS incapacitated women for an extended period of time making them unable to fulfil domestic and economic roles which were inimical to the survival of their families. Such perceptions, when widespread, have the tendency of affecting the uptake of medically indicated caesarean sections aimed at preventing perinatal and neonatal mortality, especially among rural women. The aim of this study was to explore maternal perceptions about caesarean section deliveries, and their role in reducing perinatal and neonatal mortality among antenatal attendants in selected hospitals in the Upper West Region of Ghana.

## Methods

### Study sites

The study took place at two sites within the Upper West Region: the Upper West Regional Hospital (UWRH) that is located at Wa, the regional capital which is predominantly urban, and the St. Joseph’s Hospital in the Jirapa District, which is more rural. The UWRH currently serves as a municipal and regional hospital. It is, however, the main referral centre within the healthcare delivery system in the Upper West Region, which shares a border with Burkina Faso and Cote d’Ivoire. The hospital currently has a bed capacity of 200, spread over 9 wards. The hospital’s neonatal intensive care unit (NICU) became functional at the latter part of 2016 and the hospital had a total delivery of 4915 in 2016 and 4969 in 2017. Anecdotal evidence suggests that prior to this, there had been rumours among the mothers that delivery by CS in the UWRH hospital was likely to be associated with poor maternal and neonatal outcomes. This study provides an opportunity to determine whether this prevails.

St. Joseph’s Hospital (SJH) assumes the role of a district hospital for the Jirapa District. It has seven (7) wards and this include a neonatal intensive care unit (NICU), which started operations in 2015 and was the first to be established in the Upper West Region. The total deliveries for the year 2016 were 1709 and 1663in 2017.

### Study design

This study was a cross-sectional study involving pregnant women attending antenatal clinics in two hospitals within the Upper West Region (UWR) and was conducted from 15^th^January 2018 to 29th June, 2018. It was a questionnaire-based study which examined the perceptions and experiences of pregnant women with caesarean section and the role that these perceptions and experiences play in reducing perinatal and neonatal mortality. The questionnaires were administered by trained nurses at the antenatal clinic.

### Study population

Expectant mothers were the participants in this study. All women attending antenatal clinics in the UWRH and the SJH hospitals were eligible for inclusion into the study. Pregnant women who were ill and in pain, as well as pregnant women who did not give consent were excluded from the study.

### Sampling technique and sample size

The sample size for the study was based on the assumption that the proportion of expectant mothers attending the ANC with good knowledge about caesarean section (p) is 50% (0.5). Thus at a desired confidence interval of 95% and an allowable error of 5%, a minimum sample size of 384 pregnant women attending the antenatal clinic was required. For the purpose of this study, we aimed at recruiting at least 200 participants from each study site. Using systematic sampling with antenatal registers as the sampling frame, we estimated an average of five questionnaires to be administered per day per study site within the 2 month of data collection until the desired sample size is reached. This was based on the assumption that since antenatal clinics are held on week days, 5 questionnaires a day will give us 25 in a week and 100 in a month, So that in 2 months we would obtain 200 questionnaires from each site but unfortunately the data collection took longer than we anticipated which is partly been due to cancelled clinic and holidays.

### Data collection procedure and study instrument

A structured questionnaire consisting of both closed and open-ended questions was used to assess antenatal attendants’ experiences, knowledge and perceptions towards Caesarean section (CS) delivery. The questionnaire included questions on socio-demographic information such as age, marital status, occupation, religion, ethnicity and level of education. A brief obstetric history provided information on parity, number of stillbirths or pregnancies lost and the reason for the losses. The knowledge and attitude of respondents to CS was assessed based on respondents’ response to questions such as whether they had heard about CS, reasons why some women have CS, complications of the procedure, how long someone stays in the hospital after a CS, whether blood transfusion may be required during the procedure, and whether a pregnant woman can opt for CS without medical indications. The questionnaire is attached as additional file 1. The questionnaires were pretested at the Wechiau District Hospital, which is also in the Upper West Region, before use.

### Ethical considerations

Permission to carry out this research was obtained from the Medical Directors of the Regional Hospital and St Joseph’s District Hospital. Ethical Approval was obtained from the Ghana Health Service Ethical Review Committee (GHS-ERC: 09/03/17). Written informed consent was obtained from each study participant before the administration of questionnaires. Assent was obtained from all participants less than 16 years and consent obtained from their legal guardian/parent. In carrying out the interviews, privacy and confidentiality were ensured. Participation was based entirely on goodwill and there was no compensation for participating in this study.

### Data management and analysis

A unique study number was assigned to each participant and used for the storage and management of all data relating to that patient. The data were captured using Statistical Package for Social Sciences (SPSS) version 16.0. Cleaning of data was done using standardised queries to conduct range and logic check. Discrepant entries were rectified by reviewing the record form. The data was exported into STATA version 11 (StataCorp, Texas, USA). The results were expressed as means and standard deviations (SD) for continuous variables and percentages for categorical variables such as age and sex. Statistical significance was accepted at a 5% probability level, that is, a *p*-value of less than 0.05. Cross tabulations and multiple response analysis were carried out where necessary. Logistic regression was also carried out to determine factors associated with poor perception of caesarean section among women attending the antenatal clinic.

## Results

Altogether 416 pregnant women took part in the study. Response rates observed in the study was 98%. In all, 50.5% (*n* = 210) of the expectant mothers attended antenatal clinic at St Joseph’s hospital, Jirapa, while 49.5% (*n* = 206) attended at the Upper West Regional Hospital. The age range of study participants was from 14 to 45 years with a mean age of 27 (SD = 6.0) years. Table 1 displays the background characteristics of study participants from the two study sites. With regard to the obstetric history, the current pregnancy bringing them to ANC ranged between the first to the ninth for study participants. About a third of study participants 34.2% (*n* = 142), had lost a pregnancy or had had a perinatal mortality. Among those who had had a perinatal mortality, the number of stillbirths ranged from one to a maximum of four. Table 2 displays the obstetric history of study participants and reasons ascribed for the causes of pregnancy loss or stillbirth varied. In all, 34.6% had no knowledge of the cause.

**Table 1:** Background characteristics of study participants by study site

	Hospital Attended			
Maternal Characteristics	Upper West Regional Hospital	St Joseph’s Hospital	Total	*P*-Value
	N (%)	N (%)	N (%)	
Number of Antenatal patients	206 (49.5)	210 (50.5)	416 (100)	
Age of Pregnant woman in years				0.831
19 and below	27 (13.1)	22 (10.5)	49 (11.8)	
20–24	45 (21.8)	43 (20.5)	88 (21.2)	
25–29	65 (31.6)	68 (32.4)	133 (32.0)	
30–34	42 (20.4)	51 (24.3)	93 (22.4)	
35 and above	27 (13.1)	26 (12.4)	53 (12.7)	
Highest Level of formal Education				0.001
No formal Education	21 (10.2)	53,925.6)	74 (18.0)	
Primary School	18 (8.8)	30 (14.5)	48 (11.6)	
Junior Secondary School	12 (5.9)	37 (17.9)	49 (11.9)	
Senior Secondary School	40 (19.5)	23 (11.1)	63 (15.3)	
Vocational / Technical	24 (11.7)	4 (1.9)	28 (6.8)	
Tertiary	90 (43.9)	59 (28.0)	148 (35.9)	
Occupation				0.004
Unemployed	43 (20.9)	43 (20.7)	86 (20.7)	
Trader	32 (15.5)	21 (10.1)	53 (12.8)	
Artisan	31 (15.1)	36 (17.3)	67 (16.2)	
Professional	80 (38.8)	52 (25.0)	132 (31.9)	
Student	13 (6.3)	24 (11.5)	37 (8.9)	
Other	7 (3.4)	32 (15.4)	39 (9.4)	
Religion				0.001
Christianity	119 (57.8)	180 (86.5)	299 (72.2)	
Islam	84 (40.8)	13 (6.3)	97 (23.4)	
Traditional	3 (1.5)	14 96.7)	17 (4.1)	
Others	–	1 (0.5)	1 (0.2)	
Marital Status				0.001
Never Married	37 (18.5)	7 (3.4)	44 (10.8)	
Currently Married	106 (53.0)	188 (90.8)	294 (72.2)	
Co-habiting	48 (24.0)	11 (5.3)	59 (14.5)	
Widowed	4 (2.0)	1 (0.5)	5 (1.2)	
Separated	5 (2.5)	–	5 (1.2)	
Ethnicity				0.001
Akan	24 (11.8)	10 (4.8)	34 (8.2)	
Ga/ Dangme	8 (3.9)	1 (0.5)	9 (2.2)	
Ewe	16 (7.8)	–	16 (3.9)	
Northern ethnic groups	149 (73.0)	196 (93.3)	345 (83.3)	
Non-Ghanaians	7 (3.4)	1 (0.5)	8 (1.9)	
Others	–	2 (1.0)	2 (0.5)

**Table 2:** Maternal Obstetric history

Maternal Characteristics	Upper West Regional Hospital	St Joseph’s Hospital	Total	*P*-Value
	N (%)	N (%)	N (%)	
Number of times pregnant (gravidity)				0.285
First pregnancy	64 (31.1)	67 (31.9)	131 (31.5)	
Second	46 (22.3)	64 (30.5)	110 (26.4)	
Third	37 (18.0)	27 (12.9)	64 (15.4)	
Fourth	34 (16.5)	31 (14.8)	65 (15.6)	
Fifth and above	25 (12.1)	21 (10.00	46 (11.1)	
Number of children (Parity)				0.001
None	82 (39.8)	10 (5.2)	92 (23.8)	
One	50 (24.3)	77 (42.5)	127 (32.8)	
Two	47 (22.8)	46 (25.4)	93 (24.0)	
Three	18 (8.7)	25 (13.8)	43 (11.1)	
Four and above	9 (4.4)	23 (12.7)	32 (8.3)	
History of Lost pregnancy/ Stillbirth				0.001
Yes	96 (46.6)	46 (22.0)	142 (34.2)	
No	110 (53.4)	163 (78.0)	273 (65.8)	
Number of Lost pregnancy/Stillbirths				0.041
One	62 (66.0)	38 (86.4)	100 (72.5)	
Two	24 (25.5)	5 (11.4)	29 (21.0)	
Three and above	8 (8.5)	1 (2.3)	8 (5.8)	
Causes of the pregnancy loss (multiple response analysis)				0.001
Do not know	22 (23.9)	25 (56.8)	47 (34.6)	
Infection	5 (5.4)	5 (5.4)	10 (7.4)	
Stress	5 (5.4)	5 (11.4)	10 (7.4)	
Hypertension in pregnancy	–	3 (6.8)	3 (2.2)	
Diabetes in pregnancy	–	1 (2.3)	1 (0.7)	
Malaria in pregnancy	2 (2.2)	2 (4.6)	4 (2.9)	
Spontaneous Abortion	30 (32.6)	5911.4)	41 (30.2)	
Induced Abortion	31 (33.7)	5 (11.4)	36 (26.4)	
Other reasons	11 (12.0)	1 (2.3)	12 (8.8)	

### Experience with caesarean section

A total number of 96 respondents (representing 26%) had had a previous history of Caesarean section. Table 3 displays the experiences of ANC attendants with caesarean section. The proportion of ANC attendants who had had caesarean section was higher among those attending ANC in the district hospital at 38.4% (*n* = 66) compared to those attending ANC in the regional hospital, which was at 15.7% (*n* = 30). The majority of women stated that, the indication for the previous caesarean section was “big baby’, accounting for 42.1% of the indication for caesarean section. Other reasons for the caesarean section included previous caesarean section, 26.1% (*n* = 23), baby not lying well, 25.0% (*n* = 22), and antepartum haemorrhage 4.6% (*n* = 4). Maternal request was the least, accounting for 1.1% (*n* = 1).

**Table 3:** Maternal experience with caesarean section

Maternal Characteristics	Upper West Regional Hospital	St Joseph’s Hospital	Total	*P*-values
	N (%)	N (%)	N (%)	
History of Previous caesarean section				0.001
Had a previous caesarean section	30 (15.7)	66 (38.4)	96 (26.5)	
No previous caesarean section	161 (84.3)	106 (61.6)	267 (73.6)	
Indication for previous caesarean section (multiple response analysis)				0.001
Big baby	24 (41.4)	13 (43.3)	37 (42.1)	
Previous Caesarean section	23 (39.7)	–	23 (26.1)	
Baby not lying well	7 (12.1)	15 (50.0)	22 (25.0)	
Bleeding (Antepartum Haemorrhage)	4 (6.0)	–	4 (4.6)	
Repeated Miscarriages	10 (17.2)	–	10 (11.4)	
Complicated Hypertension in pregnancy	1 (1.7)	2 (6.7)	3 (3.4)	
Maternal request	–	1 (3.3)	1 (1.1)	
Not told the reason for Caesarean section	–	2 (6.7)	2 (2.3)	
Others	6 (10.3)	5 (16.7)	11 (12.5)	
Overall description of Caesarean section experience (multiple response analysis)				0.207
Good	8 (13.6)	4 (14.8)	12 (14.0)	
Painful	39 (66.1)	18 (66.7)	57 (66.3)	
Long stay in Hospital	22 (37.3)	3 (11.1)	25 (29.1)	
Bad attitude of Staff	6 (10.2)	1 (3.7)	7 (8.1)	
Others	3 (5.1)	3 (11.1)	6 (7.0)	
Information wished to have known before the Caesarea section (multiple response analysis)				0.001
Reason for having the caesarean section	3 (5.0)	17 (58.6)	20 (22.5)	
Complications of the surgery	8 (13.3)	17 (58.6)	25 (28.1)	
Duration of stay in hospital	13 (21.7)	7 (24.1)	20 (22.5)	
Cost of the caesarean section	6 (10.0)	4 (13.8)	10 (11.2)	
Effects of medications on baby	3 (5.0)	2 (6.9)	5 (5.6)	
Other questions on caesarean section	36 (60.0)	2 (6.9)	38 (42.7)	
What would have made your caesarean section (CS) experience better (Multiple response analysis)				0.267
Education on CS at antenatal clinic	37 (75.5)	20 (71.4)	57 (74.0)	
Better staff attitude	9 (18.4)	5 (17.9)	14 (18.2)	
Shorter stay at hospital	4 (8.2)	1 (3.6)	5 (6.5)	
Been told earlier about having CS	8 (16.3)	12 (42.9)	20 (26.0)	
Experienced complication of Caesarean section (Multiple response analysis)				0.059
Bleeding	11 (18.3)	1 (3.3)	12 (13.3)	
Infection of wound	4 (6.7)	2 (6.7)	6 (6.7)	
Sick baby	6 (10)	–	6 (6.7)	
Reaction to anaesthesia medications	–	1 (3.3)	1 (1.1)	
No complications	34 (56.7)	26 (86.7)	60 (66.7)	
Other complications	8 (13.3)	3 (10.0)	11 (12.2)	
Form of Anaesthesia used for surgery				0.052
Spinal	54 (91.5)	23 (76.7)	77 (86.5)	
General	5 (8.5)	7 (23.3)	12 (13.5)	
Procedure explained before caesarean section				0.001
Procedure explained	55 (98.2)	21 (72.4)	76 (89.4)	
Procedure not explained	1 (1.8)	8 (27.6)	9 (10.6)	

With regard to the overall description of the caesarean section experience, 66.3% (*n* = 57) of participants who had a previous caesarean section described it as painful while a total of 14.0% (*n* = 12) described it as good. There was no statistical difference between the experiences of respondents who had their caesarean section at the district hospital and those who had theirs at the regional hospital (*p* = 0.267). In all, 28.1% (*n* = 25) of the pregnant women who had a previous history caesarean section wished they had information on the complications of the surgery prior to the surgery.

### Knowledge and perception of women attending antenatal clinic on caesarean section

Table 4 displays the results of the knowledge and perception of women attending ANC on caesarean section. Altogether, 364 (91%) women had heard about caesarean section. Pregnant women who had heard about caesarean section reported several reasons why women have caesarean section and these reasons are summarised in Table 4. Vaginal birth after caesarean section (VBAC) was acknowledged as possible by 77.7% (*n* = 313) of the respondents, whereas 4.5% (*n* = 18) believed that women can no longer have a vaginal delivery after a caesarean section and 17.9% (*n* = 72) stated that they had no idea about the possibility of vaginal birth after a previous caesarean section. The majority of the women interviewed preferred planned spontaneous vaginal delivery (87.4%, *n* = 348) to caesarean section though a small proportion, 3.9% (*n* = 42) of them preferred caesarean section to spontaneous vaginal delivery. A higher proportion of women with a previous caesarean section (34.5%, *n* = 30) preferred CS delivery compared to women without prior CS (3.9%, *n* = 12). In all 8.6% of all participants said they were unwilling to have caesarean section even if necessary.

**Table 4:** Knowledge and Perception of pregnant women attending antenatal clinic on caesarean section

	Previous caesarean section (CS)			
Maternal knowledge and perception	Had a Previous CS	No history of CS	Total	*P*-Value
	N (%)	N (%)	N (%)	
Study participants	89 (22.0)	315 (78.0)	404 (100.0)	
Heard of caesarean section				0.001
Heard of caesarean section	88 (100.0)	279 (89.0)	364 (91.5)	
Never heard of caesarean section	–	34 (11.0)	34 (8.4)	
Knowledge on why women have caesarean section*				0.001
Big baby	57 (64.8)	118 (69.6)	245 (68.4)	
Previous caesarean section	27 (30.7)	69 (25.6)	96 (26.8)	
Baby not lying well	37 (42.1)	130 (48.2)	167 (46.7)	
Baby in distress	24 (27.3)	73 (27.0)	97 (27.1)	
Bleeding	7 (8.0)	28 (10.4)	35 (9.8)	
Repeated miscarriages	10 (11.4)	11 (4.1)	21 (5.9)	
Complications of hypertension in pregnancy	11 (12.5)	31 (11.5)	42 (11.7)	
Complications of Diabetes in pregnancy	8 (9.1)	25 (9.3)	33 (9.2)	
Request of mother	10 (11.4)	74 (27.4)	84 (23.5)	
Other reasons	19 (21.6)	56 (20.7)	75 (21.0)	
Can a woman give birth vaginally after caesarean section				0.003
Can give birth vaginally	80 (89.9)	233 (74.2)	313 (77.7)	
Cannot give birth vaginally	4 (4.5)	14 (4.5)	18 (4.5)	
I don’t know	5 (5.6)	67 (21.3)	72 (17.9)	
Is there a need for client education on Caesarean section at antenatal clinic				0.001
There is a need	86 (96.3)	247 (79.4)	333 (83.4)	
There is no need	3 (3.4)	64 (20.6)	67 (16.8)	
Preference of planned caesarean section versus vaginal delivery				0.001
Caesarean section	30 (34.5)	12 (3.9)	42 (3.9)	
Vaginal delivery	53 (60.9)	295 (94.9)	348 (87.4)	
I don’t know	4 (4.6)	4 (1.3)	8 (2.0)	
Willingness to undergo caesarean section if the need be				0.001
Would undergo caesarean section	76 (93.8)	205 (67.4)	281 (73.0)	
Would not undergo caesarean section	2 (2.5)	31 (10.2)	33 (8.6)	
Undecided	3 (3.7)	68 (22.37)	72 (18.4)	
Reasons for not wanting to have caesarean section*				0.997
Fear of been mocked	1 (2.0)	21 (8.9)	22 (7.7)	
Fear of pain during and after surgery	21 (41.2)	114 (48.5)	135 (47.2)	
Expensive	3 (5.9)	22 (9.4)	25 (8.7)	
Long recovery time	31 (60.8)	117 (49.8)	148 (51.8)	
To avoid getting a scar	1 (2.0)	25 (10.6)	26 (9.1)	
It prevents bonding with baby	–	6 (2.6)	6 (2.1)	
Not natural	7 (13.7)	66 (28.1)	73 (25.5)	
Not God’s wish	–	20 98.5)	20 (7.0)	
Blood may be given in the process	–	25 (10.6)	25 (8.7)	
May not see my baby	1 (2.0)	5 (2.1)	6 (3.1)	
Fear of complications	19 (37.3)	86 (36.6)	105 (36.7)	
How do you see a woman who delivered by caesarean section				0.001
Normal	47 (55.3)	80 (26.0)	127 (32.3)	
Weak	14 (16.5)	143 (46.4)	157 (40.0)	
Feel sorry for her	12 (14.1)	73 (23.7)	85 (21.6)	
God’s wish	12 (14.1)	12 (3.9)	24 (6.1)	
Would you like to have caesarean section (CS) for the following reasons				0.001
CS delivery is less embarrassing	25 (49.0)	65 (50.8)	90 (50.3)	
CS allows to choose the day of birth	16 (31.4)	99 (77.3)	115 (64.3)	
Woman’s body recovers faster with CS	–	18 (14.1)	18 (10.1)	
Cs delivery is more convenient	28 (54.9)	17 (13.3)	45 (25.1)	
Is there any traditional or cultural belief that affects your preferred choice of delivery?				0.001
There is a traditional or cultural belief	14 (15.9)	10 (3.3)	24 (6.1)	
There is no traditional or cultural belief	74 (84.1)	297 (96.7)	371 (93.9)	
Factors influencing your choice of place of delivery*				0.009
Place where I attend antenatal clinic	47 (52.8)	106 (34.1)	153 (38.3)	
Skills of health workers	66 (74.2)	193 (62.1)	259 (64.8)	
Know a staff member at the health facility	2 (2.3)	8 (2.6)	10 (2.5)	
Proximity to where I live	8 (9.0)	41 (13.2)	49 (12.3)	
Other reasons	6 (6.7)	36 (11.6)	42 (10.5)	
Is there any facility you would like to avoid				0.001
There are facilities I would avoid	26 (29.2)	21 (6.8)	47 (11.8)	
No facility that I would avoid	63 (70.1)	290 (93.3)	353 (88.4)	
Can having caesarean section (CS) adversely affect your child				0.239
CS can adversely affect my child	16 (18.0)	69 (22.6)	85 (21.6)	
CS has no effect on my child	56 (62.9)	161 (52.8)	217 (55.1)	
Don’t know	17 (19.1)	75 (24.6)	92 (23.4)	
Can having caesarean (CS) section promote child survival				0.001
CS promote child survival	73 (82.0)	146 (47.1)	219 (54.9)	
CS does not promote child survival	6 (6.7)	43 (13.9)	49 (12.3)	
I don’t know	10 (11.2)	121 (39.0)	131 (32.8)	

*multiple response analysis

With regard to the effect of CS on child survival, 55.1% (*n* = 217) stated that CS has no effect on their child’s survival. However, 21.6% (*n* = 85) and 23.4% (*n* = 92) of women respectively stated that “CS can have adverse effect on child survival” and “I don’t know the effect of CS on child survival”. Likewise 54.9% (*n* = 219) perceived that CS can promote child survival while 12.3% (*n* = 49) stated that CS does not promote child survival. Among those women who had previous caesarean section, 6.7% (6/90) reported sick baby as complications to CS. Also, 28.7% (112/403) of all pregnant women in the study believed that CS causes injury to baby.

Factors associated with poor perception of caesarean section among women attending ANC.

Tables 5 and 6 display the factors associated with poor perception of caesarean section among women. Pregnant women aged 19 years and below are more likely to have poor perception of caesarean section (OR = 2.5, 95% CI =1.0–6.5) when compared to those aged 35 years and above. Highest educational status significantly affected perception of caesarean section among pregnant women attending ANC. Compared with pregnant women who completed a tertiary education, having no formal education was 2.2 (95% CI = 1.2–4.1) times more likely to be associated with poor perception of CS. With regard to occupational status, the professionals were less likely to have poor perception of CS when compared to the unemployed (OR = 0.4, 95% CI = 0.2–0.7).

**Table 5:** Crude odds ratio of factors associated with poor perception of caesarean section among pregnant women attending antenatal clinic

Maternal characteristics	Crude Odds Ratio	95% Confidence interval	*P*-Value
Age of Pregnant woman in years			
19 and below	2.5	1.0–6.5	0.06
20–24	1.5	0.7–3.1	0.3
25–29	1.1	0.5–2.1	0.8
30–34	0.6	0.3–1.3	0.2
35 and above	–		
Highest Level of formal Education			
No formal Education	2.2	1.2–4.1	0.01
Primary School	2.7	1.3–5.7	0.01
Junior Secondary School	3.0	1.4–6.7	0.004
Senior Secondary School	2.3	1.2–4.4	0.01
Vocational / Technical	1.9	0.8–4.6	0.2
Tertiary	–		
Occupation			
Unemployed	–		
Professional	0.4	0.2–0.7	0.002
Others example: artisans, traders etc	0.7	0.4–1.2	0.2
Number of children (Parity)			
None	–		
One	0.4	0.2–0.7	0.002
Two	0.4	0.2–0.8	0.01
Three	0.3	0.2–0.8	0.01
Four and above	1.0	0.4–2.8	0. 9
Willingness to undergo CS if the need be			
Yes	–		
No or undecided	4.4	2.4–8.3	0.0001
Is there any facility you would avoid for child delivery			
No	–		
Yes	0.3	0.2–0.60	0.0003
Having traditional or cultural belief on choice of delivery method			
No traditional or cultural belief	–		
Have a traditional or cultural belief	0.4	0.2–0.9	0.02
Preference of planned caesarean section versus vaginal delivery			
Prefer planned caesarean section	–		
Prefer Spontaneous vaginal delivery	7.3	3.7–14.4	0.0001
Study site: Currently attending Antenatal clinic in a regional versus district hospital			
Regional (Upper West regional hospital, Wa)	2.0	1.3–3.0	0.002
District (St Joseph’s hospital, Jirapa)	–		
History of a previous caesarean section			
No previous caesarean section	3.5	2.1–5.7	0.0001
Had a previous caesarean section	–		
Preferred mode of delivery			
Caesarean section	–		
Vaginal delivery	3.7	1.8–7.8	0.0004

**Table 6:** Multivariate logistic regression of factors independently associated with poor perception of caesarean section among pregnant women attending antenatal clinic; adjusting for education status, age and religion

Maternal characteristics	Crude Odds Ratio	95% Confidence interval	*P*-Value
Willingness to undergo CS if the need be			
Yes	–		
No or undecided	3.8	2.0–7.3	0.001
Is there any facility you would avoid for child delivery			
No	–		
Yes	0.4	0.2–0.8	0.005
Preference of planned caesarean section versus vaginal delivery			
Prefer planned caesarean section	–		
Prefer Spontaneous vaginal delivery	6.3	3.0–13.4	0.001
Study site: Currently attending Antenatal clinic in a regional versus district hospital			
Regional (Upper West regional hospital, Wa)	3.0	1.7–5.2	0.000
District (St Joseph’s hospital, Jirapa)	–		
History of a previous caesarean section			
No previous caesarean section	2.4	1.4–4.1	0.001
Had a previous caesarean section	–		
Preferred mode of delivery			
Caesarean section	–		
Vaginal delivery	3.0	1.3–6.7	0.007

Pregnant women who were undecided or unwilling to undergo caesarean section were 4.4 times (95% CI = 2.4–8.3) more likely have poor perception about caesarean section. Also pregnant women who preferred planned spontaneous vaginal delivery compared to CS were 7.3 (95% CI = 3.7–14.4) times more likely to have poor perception of CS. Other factors that were independently associated with the poor perception of caesarean section included the following: no previous caesarean section history (OR = 3.5, 95% CI = 2.1–5.7). In a model adjusting for maternal age, educational status and religion, the following factors remain significantly associated with poor perception of caesarean section: undecided or not willing to undergo caesarean section (OR = 3.8, 95% CI = 2.0–7.3), preference for spontaneous vaginal delivery compared to planned caesarean section (OR = 6.3, 95% CI =3.0–13.4), attending ANC in a regional hospital (OR = 3.0, 95% CI = 1.7–5.2), having no history of a previous caesarean section (OR = 2.4, 95% CI = 1.4–4.1), and having vaginal delivery as the preferred mode of delivery (OR = 3.0, 95% CI = 1.3–6.7).

Factors associated with pregnancy loss/ perinatal mortality among women attending ANC.

Table 7 displays factors associated with perinatal mortality among women attending ANC. Comparing those with a history of caesarean section with women without history of CS, positive CS history was 4.6 times (95% CI 2.7–7.8) more likely to be associated with likelihood of having a history of pregnancy loss or perinatal mortality. Also adjusting for educational status and religion, women having a traditional or religious belief against CS were 3.2 (1.3–7.9) times more likely to have a pregnancy/ perinatal mortality compared with pregnant women without such belief. Women attending ANC in the district hospital were less likely to have had a pregnancy loss or perinatal mortality compared with women attending ANC in the regional hospital (AOR = 0.4, 95% CI 0.2–0.6).

**Table 7:** Factors associated with pregnancy loss/ perinatal mortality among women attending ANC

Maternal characteristics	Adjusted Odds Ratio	95% Confidence interval	*P*-Value
Age of Pregnant woman in years			
19 and below	–		
20–24	2.7	1.0 – 7.7	0.05
25–29	3.2	1.2–8.8	0.02
30–34	5.1	1.8–14.4	< 0.005
35 and above	5.3	1.9–15.9	< 0.005
Number of times pregnant (gravidity)			
Two and below	–		
Three	8.4	4.3–16.3	< 0.001
Four	14.3	7.2–28.3	< 0.001
Five and above	15.7	6.8–36.4	< 0.001
Number of children (Parity)			
Zero – one	–		
Two and above	1.9	1.2–3.0	0.005
History of previous caesarean section			
No previous caesarean section			
Positive history of previous caesarean section	4.6	2.7–7.8	< 0.001
Having traditional or cultural belief against having caesarean section			
No traditional or cultural belief	–		
Have a traditional or cultural belief	3.2	1.3–7.9	0.01
Preference of planned caesarean section versus vaginal delivery			
Prefer planned caesarean section	–		
Prefer Spontaneous vaginal delivery	0.4	0.2–0.8	0.005
Study site: Currently attending Antenatal clinic in a regional versus district hospital			
Regional (Upper West regional hospital, Wa)	–		
District (St Joseph’s hospital, Jirapa)	0.4	0.2–0.6	< 0.001
Form of anaesthesia used in the previous caesarean section			
Spinal Anaesthesia	–		
General Anaesthesia	0.2	0.05–0.9	0.04

## Discussion

Globally, caesarean section rates increase with increasing socio-economic development and are a proxy measure for assessing progress in maternal and infant health [24, 25]. Thus one would have expected that the CS rate would be higher among women attending ANC at the regional hospital as has been reported at the Korle Bu Teaching Hospital [18]. However in this study, the proportion of ANC attendants with CS experience from the district hospital was more than twice the proportion of CS experience in the regional hospital. Data from this study showed that participants in the district hospital had a greater number of pregnancies and children compared with the regional hospital for the same comparative maternal age. Since the proportion of CS increases with the number of pregnancies, the observed higher proportion of CS among those in the district hospital can be attributed to the differential number of births or pregnancies between the two groups.

Majority of the women preferred spontaneous vaginal delivery to caesarean section though a small proportion of women preferred caesarean section to spontaneous vaginal delivery. This is not an unexpected finding and is similar to a study in Ghana which found that 55% of pregnant women indicated similar preference [26]. Other studies in Ghana found 93.3% [27] and 94% [28] and a study from Nigeria [20] reported 85.7%. This preference for vaginal delivery has been described as a reflection of the desire to have a natural birthing process rather than an aversion for CS [27], an attitude also portrayed by midwives and obstetricians in Sweden, a developed country with low CS rates [29]. There is worldwide deliberation about the significance of CS performed without medical indication [24, 25]. However, in this part of the world, maternal request due to non-medical indication seems to be of low priority. The limited access to health facilities with the capacity to conduct major surgeries, coupled with increased workload on the few trained health professional with the requisite skill, makes maternal request for caesarean section a difficult request to satisfy [30]. Also, traditional and cultural practices that discourage operative delivery, most likely contribute to the low number of CS for non-medical reasons [19, 31]. However, contrary to the current findings, a recent report indicates that CS without medical indication is gaining ground in some centres in Africa [30].

Respondents generally had inadequate knowledge about CS, a finding which has been attested in other studies [20, 28]. This is contrary to a study from Nigeria which found good knowledge among respondents [19]. The indications for CS were also not well known. This is similar to findings from a study in Cape Coast, Ghana, which found that only 45% of respondents knew at least one indication of CS [28]. Also, a substantial proportion of respondents believed that CS can have adverse effects on child survival and does not promote child survival. Almost half (45.1%), consisting mostly of those who had never had a CS, did not know or feel that CS can promote child survival and one fifth of them (21.6%) believed that CS can have adverse effects on child survival. This is similar to the findings of other studies [19, 27]. Over half of the respondents did not seem to be aware of indications for CS that prevent foetal compromise such as foetal distress and abnormal lie. Thus educative messages on CS should emphasise its role in protecting the foetus.

As expected, the mothers who had had a previous CS seemed generally more knowledgeable and well informed about CS than those with no prior CS experience [19, 28]. However, women in this study indicated that they had had several unanswered questions prior to the surgical procedure and if these had been addressed by health professionals before the surgery, it would have improved the CS experience. This suggests inadequate counselling prior to caesarean section among study the participants. Deliberate efforts should be made to educate pregnant women on CS and these efforts can be supported by the use of leaflets, posters, DVD’s and case studies of mother who have had previous CS [28]. This can be delivered by a special counselling team of dedicated midwives as is done in Sweden to allay the fears of women [29]. It can also be a part of the general education given at school on childbirth as studies have also found that the level of knowledge about CS is influenced by one’s level of education [19, 28], though others did not find a positive association [32]. A positive finding from this study is that the majority of respondents believed that adequate counselling and education during antenatal care might prepare women adequately for CS and are likely to increase the chances of success [27].

The majority of women were willing to have caesarean section if only if the need arose which is similar to findings in other more urban parts of the country and Nigeria [19, 27, 28]. Some women however would not undergo CS for any reason. This finding is similar to findings from a community-based study in the Upper West Region and one from Burkina Faso [33] and studies from Nigeria and Ghana which reported fear of death and pain as the main dangers [19, 20]. The Upper West Region shares a border with Burkina Faso, and the regional hospital has reported cases of maternal mortality from Burkina Faso so it is not surprising that these studies have similar findings [25, 33]. Long recovery time is important in these environments since many of the rural women are poor subsistence farmers with several domestic roles. Therefore, the perception that CS weakens women, thereby limiting their physical capacity to farm, carry water or do other work, and restricts their ability to generate income for their families, may make them want to avoid institutional delivery [21, 25]. This delay may be aggravated by the lack of transport facilities and poor roads which are prevalent in the region and which most likely contribute to high maternal deaths in the region [34, 35].

Several other reasons for not wanting a CS were identified in this study. Some of them are fear of pain, fear of complications, CS not being a natural procedure, fear of being mocked and the avoidance of a scar reported among others. These findings are similar to reports from other studies [19, 33]. These fears may prevent women from delivering in hospitals when they perceive a higher chance of CS and they may delay presentation for emergency obstetric care required at the individual maternal level to prevent perinatal and neonatal mortality [30]. Thus measures to improve the uptake of emergency obstetric care must give consideration to how to address these fears and socio-economic factors surrounding CS during the recovery period [21, 25, 33].

This study has some limitations. All participants in the study were from the Upper West Region and as such the findings may not be representative of the entire country, Ghana. There were variations with respect to the location and characteristics of the attendants to the facilities. Also being hospital-based study, the findings from the study may not entirely reflect the perceptions of those who deliver at home or in primary care facilities.

## Conclusion

The majority of the respondents preferred vaginal delivery in this study and the reasons provided centred on long recovery time from CS, fears, attitudes, values, traditional and cultural beliefs. Though respondents generally had inadequate knowledge about CS, they had a positive attitude towards the uptake of CS. However, almost half of women did not know or feel that CS can promote child survival. This needs to be addressed through education to improve the perception of CS as a promoter of child survival and to discourage the notion that it causes adverse perinatal or neonatal outcome, particularly in at risk populations. It requires deliberate effort by midwives to present CS as an option for childbirth and improved neonatal survival which can be incorporated into general education on childbirth at antenatal clinics, in schools as well as public education. Additional effort should be made to reduce the recovery time and provide support systems for those who have a CS in this setting; otherwise the negative perceptions are likely to persist.

## Supplementary information

**Additional file 1. **Questionnaire, maternal perceptions about caesarean section & neonatal health questionnaire.

## Data Availability

The datasets used and/or analyzed during the current study are available from the corresponding author on reasonable request.

## Abbreviations

ANC: Antenatal Clinic
CS: Caesarean Section
NICU: Neonatal Intensive Care Unit
SDG: Sustainable Development Goal
UWRH: Upper West Regional Hospital
VBAC: Vaginal Birth after Caesarean Section
