# Supplementary material for: Maternal perceptions about caesarean section deliveries and their role in reducing perinatal and neonatal mortality in the Upper West Region of Ghana; a cross-sectional study
Source: BMC Pregnancy Childbirth. 2019 Oct 11;19:350. doi: 10.1186/s12884-019-2536-8 (PMC6788025; doi:10.1186/s12884-019-2536-8)
Supplement: Supplementary file 1 — Additional file 1. Questionnaire, maternal perceptions about caesarean section & neonatal health questionnaire. [file 12884_2019_2536_MOESM1_ESM.docx]

**QUESTIONNAIRE MATERNAL PERCEPTIONS ABOUT C/S & NEONATAL HEALTH**

**STUDY SITE: 1. UWR HOSPITAL 2.ST JOSEPH’S HOSPITAL**

**CODE NO: ________________**

**SECTION A SOCIO-DEMOGRAPHIC CHARACTERISTICS**

Please tick the appropriate option and/or fill in the blank spaces

1. How old are you?_____________________
2. What is your highest level of educational? a) No formal Education b) Primary c) JHS d) SHS e) Vocational/Technical f) Tertiary g) Other (specify)....................
3. What is your Occupation? a) Unemployed b) trader c) artisan d) professional e) student f) other (specify)....................
4. What is your Religion? a) Christianity b) Islam c) Traditional d) Other (specify)...............
5. What is your marital status? a) Never married b) currently married c) co- habiting d) Widowed e) Separated
6. What is your Ethnicity a) Akan b) Ga c) Ewe d) Northern tribe e) non- Ghanaian f) Other (Specify)________________
7. How many children do you have? _____________________________________
8. How many times have you been pregnant?_______________________________
9. Have you lost any pregnancy or had a stillbirth? a) yes b) no
10. If your answer to question 9 is YES, how many pregnancy losses or stillbirths have you had?

________________________

1. What was/were the cause(s)? (Please tick as many as applicable)
2. I don’t Know
3. Infection
4. Stress
5. Hypertension in pregnancy
6. Diabetes in Pregnancy
7. Malaria
8. Miscarriage
9. Abortion
10. I did not want pregnancy
11. Abortion
12. I did not want pregnancy
13. Other (specify)____________________

**SECTION B: KNOWLEDGE OF CAESAREAN SECTION**

12. Have you heard of Caesarean section (CS)? a) Yes b) No

13. If your answer to question 12 is YES, why do some women have CS? (multiple answers allowed)

- 1. Big baby
  2. Previous CS
  3. Baby not lying well
  4. Baby in distress
  5. Bleeding
  6. Repeated miscarriages
  7. Complications from hypertension in pregnancy
  8. Complications from Diabetes in pregnancy
  9. Request of mother
  10. Other (specify) 1. _______________________2. __________________

14. Have you had a CS before? a) Yes b) No

If your answer to question 14 is YES, please answer questions 15- 22. If your answer is NO,

please proceed to question 23

15. What was the reason?

a) Big baby

b) Previous CS

c) Baby not lying well

d) Bleeding

e) Repeated miscarriages

f) Complications from hypertension in pregnancy

g) Complications from Diabetes in pregnancy

h) Request of mother

i) I was not told

j) Other (specify)......................................

16. Where did you have it? _______________________

17. How would you describe the experience? a) Good b) Painful c) Long stay in hospital d) Bad attitude from staff e) Other (specify)_______________________

18. Is there any information that you wished you had known before the CS that would have made

it better? (Please tick as many as applicable)

a) Reason for having the CS

b) Complications of the surgery

c) Duration of stay in hospital

d) Cost of CS

e) Effect of medications on baby

f) Other (specify)..................................................................................

19. What would have made the experience better? (Please tick as many as applicable)

a) Education on CS at antenatal clinic

b) Better staff attitude

c) Shorter stay at hospital

d) Been told earlier about having CS

e) Others (specify)......................................................................

20. Did you experience any complications? (Please tick as many as applicable)

a) No complication

b) Bleeding

b) Infection of wound

c) Sick baby

d) Reaction to medication (anaesthesia)

e) Other (specify).....................................................

21. What form of medication (anaesthesia) was used?

a) Spinal (Saw my baby when it was brought out)

b) General (did not see my baby, I was asleep)

22. Was the procedure explained to you before the Operation? a) Yes b) No

23. What are the complications associated with CS delivery (Please tick as many as applicable)

a) Bleeding

b) Injury to body parts

c) Injury to baby

d) Infection of wound

e) Big abdomen

f) CS delivery in next pregnancy

g) Other (specify)...............................

24. How many days will someone who has a CS stay in hospital?________________

25. Can a woman give birth vaginally after a CS? a) Yes b) No c)I don’t know

26. If your answer to question 25 is NO, why would women who have CS not be able to give

birth vaginally?________________________________________________________________

_____________________________________________________________________________

27. Do you think there is a need for client education on Caesarean section at the antenatal clinic?

a) Yes b) No

28. If your answer to question 27 is YES, what would you want to know about CS?___________

______________________________________________________________________________

29. Would you prefer planned Caesarean or vaginal delivery? a) CS b) vaginal delivery c) I don’t know.

30. Why do you prefer this choice? ___________________________________________

31. Are you willing to undergo CS if the need be? a) Yes b) No c) undecided d. Other________

32. Why would you not want to have a CS?

a) Fear of been mocked

b) Fear of pain during and after surgery

c) Expensive

d) Long recovery time

e) To avoid getting a scar from CS

f) Prevents bonding with baby

g) Not natural

h) Not God’s wish

i) Blood may be given in the process

j) I may be asleep during the procedure (general anaesthesia) and may not see mybaby

k) Fear of complications

33. How do you see a woman who has delivered by CS? a) Normal b) Weak c) Feel sorry for herd) God’s wish) Other (specify) .......................................

34. Would you like to have a CS for any of the following reasons (Multiple answers allowed)

a. Having babies by CS is less embarrassing than having them vaginally

b. A CS birth is better because it allows one to choose the day of the baby’s birth

c. A woman’s body recover faster from CS compared with a vaginal birth

d. A CS delivery is more convenient

e. Other (specify).....................................................................................................

35. Is there any tradition or cultural belief that affects your preferred choice of delivery?

a) Yes

b) No

36. If your answer to question 35 is YES, what is it__________________________________

............................................................................................................................................................

............................................................................................................................................................

37 What is your preferred mode of delivery if you had a choice?________________

38 Where would you like to have your baby? _______________________________

39 Reason for delivering there 1. Antenatal Attendant 2. Skill 3. Know someone there 4. Close by where I live 5. Other (specify)___________________________

40 Is there a facility you will want to avoid 1.Yes (specify)________________ 2. No

41 If your answer to Qu 40 is yes specify reason___________________________

42. Can having a Caesarean section promote the child survival? 1. Yes 2. No 3. Don’t Know

43. If your answer is yes in what way does a Caesarean section promote child survival?

______________________________________________________________________________

______________________________________________________________________________

44. Can having a Caesarean section adversely affect your child? 1. Yes 2. No 3. Don’t Know

43. If your answer is yes in what way can a Caesarean section adversely affect your child?

______________________________________________________________________________

______________________________________________________________________________
